# *Akkermansia muciniphila* isolated from forest musk deer ameliorates diarrhea in mice via modification of gut microbiota

**DOI:** 10.1002/ame2.12441

**Published:** 2024-06-03

**Authors:** Yan Deng, Yan Wang, Ying Liu, Xiaoli Yang, Hai Zhang, Xiaochang Xue, Yi Wan

**Affiliations:** ^1^ Key Laboratory of the Ministry of Education for Medicinal Resources and Natural Pharmaceutical Chemistry College of Life Sciences, Shaanxi Normal University Xi'an China; ^2^ Shaanxi Institute of Microbiology Xi'an China; ^3^ NMPA Key Laboratory for Testing Technology of Pharmaceutical Microbiology, Shaanxi Institute for Food and Drug Control Xi'an China; ^4^ Department of Cell Biology, National Translational Science Center for Molecular Medicine Fourth Military Medical University Xi'an China

**Keywords:** *Akkermansia muciniphila*, diarrhea, enterotoxigenic *Escherichia coli*, forest musk deer, gut microbiota

## Abstract

**Background:**

The forest musk deer, a rare fauna species found in China, is famous for its musk secretion which is used in selected Traditional Chinese medicines. However, over‐hunting has led to musk deer becoming an endangered species, and their survival is also greatly challenged by various high incidence and high mortality respiratory and intestinal diseases such as septic pneumonia and enteritis. Accumulating evidence has demonstrated that *Akkermannia muciniphila* (AKK) is a promising probiotic, and we wondered whether AKK could be used as a food additive in animal breeding programmes to help prevent intestinal diseases.

**Methods:**

We isolated one AKK strain from musk deer feces (AKK‐D) using an improved enrichment medium combined with real‐time PCR. After confirmation by 16S rRNA gene sequencing, a series of in vitro tests was conducted to evaluate the probiotic effects of AKK‐D by assessing its reproductive capability, simulated gastrointestinal fluid tolerance, acid and bile salt resistance, self‐aggregation ability, hydrophobicity, antibiotic sensitivity, hemolysis, harmful metabolite production, biofilm formation ability, and bacterial adhesion to gastrointestinal mucosa.

**Results:**

The AKK‐D strain has a probiotic function similar to that of the standard strain in humans (AKK‐H). An in vivo study found that AKK‐D significantly ameliorated symptoms in the enterotoxigenic *Escherichia coli* (ETEC)‐induced murine diarrhea model. AKK‐D improved organ damage, inhibited inflammatory responses, and improved intestinal barrier permeability. Additionally, AKK‐D promoted the reconstitution and maintenance of the homeostasis of gut microflora, as indicated by the fact that AKK‐D‐treated mice showed a decrease in *Bacteroidetes* and an increase in the proportion of other beneficial bacteria like *Muribaculaceae*, *Muribaculum*, and unclassified *f_Lachnospiaceae* compared with the diarrhea model mice.

**Conclusion:**

Taken together, our data show that this novel AKK‐D strain might be a potential probiotic for use in musk deer breeding, although further extensive systematic research is still needed.

## Graphical abstract

The forest musk deer is a rare fauna species in China famous for its musk secretion, which is used in selected Traditional Chinese medicines, but excessive hunting and high mortality from respiratory and intestinal diseases like septic pneumonia and enteritis make musk deer an endangered species. *Akkermansia muciniphila* (AKK) isolated from musk deer can improve enterotoxigenic *Escherichia coli* (ETEC)‐induced diarrhea in mice by regulating the reconstitution of intestinal flora and homeostasis, hence expanding the potential probiotic functions of AKK which could be used as a potential probiotic for musk deer breeding.

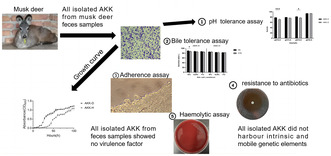

## 1 INTRODUCTION

The forest musk (*Moschus* spp.) is a rare fauna species in China renowned for its musk secretion.^1^, ^2^ Excessive hunting has led to the endangered status of this particular genus.^3^ To safeguard musk resources, nature reserves have been built to strengthen conservation of wild musk deer populations and their habitats, with artificial breeding as a significant and successful supplement.^4^ However, respiratory and intestinal diseases like pneumonia and enteritis occur frequently and are often fatal among artificially reared forest musk, which greatly challenges their long‐term survival.^5^ Although antibiotics can be used to treat infectious diseases, the excessive use of antibiotics has led to problems^6^ like heightened drug resistance, antibiotic residues, the emergence of superbugs, and environmental contamination.^7^

Probiotic preparations can alleviate the adverse effects associated with the use of antibiotics and enhance the immune function of musk deer^8^; they also improve the composition and microecological environment of animal gut flora, establish a microbiological barrier primarily populated by probiotics, alleviate the imbalance of microbiota, and thus prevent the infiltration and proliferation of harmful and transient bacteria. These effects result in improved feed intake and conversion efficiency in livestock and poultry, as well as a reduction in the incidence of diseases and mortality rates.^7^, ^9^ However, there is currently no probiotic formula available for the dietary needs of forest musk.

Currently, there are 34 permitted feed additive species. The use of novel probiotic strains, including *Akkermannia muciniphila* (AKK),^10^ *Bacteroidaceae* spp.,^11^, ^12^ *Clostridium* spp.,^13^ and *E. faecalis*,^14^ has gained popularity in the field of probiotics due to their demonstrated efficacy in disease prevention and therapy.

AKK is a bacterium known for its ability to degrade mucus in the intestinal mucosa. During this degradation process, it releases amino acids and monosaccharides, as well as short‐chain fatty acids that serve as an energy source for the commensal flora residing in the intestinal tract. Thus, AKK can regulate the composition of the intestinal flora. Emerging studies have provided compelling evidence of the participation of AKK in various clinical and physiological processes. In 2021, pasteurized AKK was proposed for use as a food ingredient.

In light of the robust regulatory function of AKK in intestinal immune homeostasis via modulation of commensal flora, it may be a suitable probiotic for feeding to musk deer. Here, we isolated AKK from musk deer, evaluated its probiotic function, and verified its protective effects on an enterotoxigenic *Escherichia coli* (ETEC)‐induced mouse diarrhea model. Our findings suggested that AKK has the potential to be used for the prevention and treatment of musk deer diarrhea.

## 2 METHODS

### 2.1 Sample collection, isolation and purification of AKK

Musk deer feces (*n* = 20) were collected in The Feng County Huang Forest Musk Deer Breeding Center, stored in sterile centrifuge tubes, and sealed to prevent cross‐contamination, and promptly analyzed upon arrival. The samples were homogenized (10% w/v) in a sterilized phosphate buffer, diluted in sterile saline, followed by enrichment culture at 37°C under anaerobic conditions. The synthetic nutrient medium was supplemented with sterile rumen fluid (containing KH_2_PO_4_, Na_2_HPO_4_, NH_4_Cl, NaCl, MgCl_2_·6H_2_O, CaCl_2_, alkaline trace element solution, acid trace element solution, vitamin solution, resazurin, NaHCO_3_, and Na_2_S·7–9H_2_O) and pig gastric mucin‐type III. L‐Cysteine 0.05% (w/v) (Sigma‐Aldrich, St Louis, MO, USA) was used to remove a trace amount of O_2_ from the medium. The samples obtained from the enrichment bottles were then subjected to plating on Brain Heart Infusion (BHI) agar, supplemented with 0.05% (w/v) L‐cysteine and pig gastric mucin (0.5%, Type III) and some assistant prebiotics for AKK strains. The plates were placed in an anaerobic chamber (ELECTROTEK) and incubated at 37°C for 48 h. Finally, colonies exhibiting distinct morphological characteristics were carefully chosen and subsequently streaked onto an agar plate to obtain samples of pure isolates.

### 2.2 Bacterial strains and culture

AKK was cultivated in 10 mL BHI medium supplemented with mucin (4 g/L) under anaerobic conditions achieved by providing a gas phase consisting of 1.8 atm of N_2_ and CO_2_ at a ratio of 80/20 (v/v). For mouse oral gavage delivery, the AKK was diluted using anaerobic PBS to 2 × 10^10^ colony‐forming units (CFU) in 0.2 mL and administered daily.^15^ To establish the murine diarrhea model, ETEC was cultivated in a liquid broth medium at 37°C overnight. After collection by centrifugation at 5000 r/min for 5 min, ETEC was washed three times using PBS, and resuspended in PBS to a final concentration of 2.75 × 10^8^ CFU/mL.^16^

### 2.3 Identification of AKK by 16S rRNA sequencing

The genomic DNA of AKK was extracted using a bacterial DNA isolation reagent (Sangon, Shanghai, China). Then, the 16S rRNA gene and the specific DNA sequence of AKK were amplified by universal PCR primers: 27F (5'‐AGAGTTTGATCCT GGCTCAG‐3') and 1492R (5'‐TACGGCTACCTTGTTACGACT‐3'), and the specific primers of AKK AM1 (5'‐CAGCACGTGAAGGTGGGAC‐3') and AM2 (5'‐CCTTGCGGTTGGCTTCAGAT‐3'),^17^ with the parameters set as follows: 95°C for 10 min; followed by 30 cycles of denaturation at 94°C for 30 s, annealing at 60°C for 30 s, elongation at72°C for 1.5 min; and thermal retardation at 72°C for 10 min. PCR products were sequenced at the Sangon Biotech Co., Ltd (Shanghai, China) and subjected to the National Center for Biotechnology Information's (NCBI) Basic Local Alignment Search Tool (BLAST).^18^

### 2.4 In vitro screening of the probiotic potential of AKK‐D

The acid and bile salt tolerance,^19^ resistance to stimulated gastrointestinal tract juices,^20^, ^21^ adhesion to hydrocarbons,^22^, ^23^ auto‐aggregation,^22^ biofilm forming,^24^ and adhesion to intestinal epithelial cells (IECs)^19^, ^25^ of the AKK‐D strain were determined using standard methods, with AKK‐H used as a control.

### 2.5 Safety evaluation of isolates

For the safety evaluation, AKK‐D was assessed for antibiotic susceptibility,^26^ hemolytic activity,^21^ and its ability to produce biogenic amines^27^ using routine protocols. AKK‐H was used as a parallel control.

### 2.6 Animal experiments

#### 2.6.1 Diarrhea model establishment

Male specified pathogen‐free (SPF) C57BL/6 mice (8 weeks old) were obtained from Shaanxi Normal University and were maintained in a controlled environment (23 ± 2°C, 40%–60% humidity, and a 12 h light/dark cycle). After an adaptation period of 1 week, mice were randomly divided into 3 groups: Control group (basal diet), ETEC model group (basal diet with ETEC challenge), and ETEC model + AKK group (Oral gavage AKK with ETEC challenge). The mice were fed with basal diet/AKK for 14 days. On the 15th day, mice were intraperitoneally injected with 0.1 mL PBS or ETEC solution (2.75 × 10^8^ CFU/mL) per 10 g body weight. Three days after the last administration, mice were sacrificed and serum, and liver, spleen, and colon tissues were collected. Diarrhea indices were recorded, tissue morphology was analyzed, and serum cytokines (IL‐6, IL‐10, and TNF‐α) were measured.^28^ Fresh feces were collected for bacterial burden determination.

#### 2.6.2 Water content of stool

Stool water content was measured as a marker of diarrhea according to a previously described procedure.^29^, ^30^ Briefly, feces were collected every 3 days and immediately weighed to define ‘wet weight’. After incubated at 37°C for 72 h, feces were weighed to define ‘dry weight’. The water content in stool was expressed as a percentage of (wet weight−dry weight)/wet weight and normalized to water content in uninfected mice. Dried samples were stored frozen until they were analyzed for chloride content.

#### 2.6.3 Scoring of diarrhea severity

The diarrhea severity was evaluated according to a previously described method.^30^, ^31^ The scoring system consisted of five grades. Grade 1, regular colon with well‐formed feces in the distal colon or rectum. Grade 2, white discoloration and gas bubbles in the lumen of cecum, accompanied by a reduced size. Grade 3, cases with severe typhlitis, characterized by a white, shrunken cecum, together with the presence of a fluid‐filled proximal colon and properly formed feces in the distal colon. Grade 4, a condition characterized by grade 3 plus fluids throughout most of the colon, accompanied by soft feces in the distal colon. Grade 5, a white and shrinking cecum, along with a distal colon containing fluid and minimal to no solid feces.

#### 2.6.4 ELISA analysis

The levels of inflammatory cytokines like IL‐6, IL‐1β, and TNF‐α in serum were measured with ELISA Kits according to the manufacturer's instructions (Jiancheng, Nanjing, China).^32^

#### 2.6.5 Histological analysis

To evaluate the extent of infection, Hematoxylin and Eosin (H&E) staining was performed. In brief, colon tissue was obtained and preserved in a 4% (v/v) paraformaldehyde (PFA) solution, and slices were affixed onto glass slides and subjected to H&E staining to facilitate histologic assessment, employing established criteria as described.^28^ The degree of submucosal edema, maintenance of epithelial integrity, retention of goblet cells, and infiltration of polymorphonuclear cells into the lamina propria were evaluated microscopically. Each category was assigned a score ranging from 0 to 4. The cumulative pathological scores recorded for each sample are reflected in the overall scores. There are three distinct levels of inflammation, namely severe, moderate, and mild inflammation, and absence of inflammation with scores of 12, 8–12, 4–8, and 4, respectively.

#### 2.6.6 Immunofluorescence staining

The colon segment was fixed using 4% PFA, dehydrated in ethanol gradients, and permeabilized in a PBS solution containing 0.1% Tween 20. Subsequently, immunofluorescence staining was performed with a kit from Beyotime (Shanghai, China) according to the manufacturer's protocol. Briefly, sections were washed with washing solution and blocked for 1 h using the blocking solution. Next, the colon segment was incubated with primary antibodies against ZO‐1 (1:250; Abclonal) and Occludin (1:250; Proteintech) overnight at 4°C, followed by incubation with secondary antibodies. The cell nuclei were stained with DAPI. Finally, images were captured with a fluorescence microscope.^33^

#### 2.6.7 RNA isolation and gene expression

RNA was extracted from colon tissues with Trizol and cDNA was generated by the PrimeScript™ Reverse Transcriptase Kit (Takara, Kusatsu, Japan). The StepOne Real‐Time PCR System was used to conduct qPCR, using a ChamQ Universal SYBR qPCR Master Mix (Vazyme, Nanjing, China). The quantification of gene expression was performed using the 2^−∆∆CT^ method, with endogenous GAPDH as a reference control.^34^ The primers are listed in Table S1.

#### 2.6.8 Bacterial diversity analysis

Stool samples were freshly collected from mice in three independent experiments and stored at −80°C until use. Three stool samples were randomly selected from each group and were used for gut microbiota analysis. More details are presented in the Supplementary Materials section.

### 2.7 Statistical analysis

All data were subjected to analysis of variance (ANOVA) using GraphPad Prism 8.0 software, and a *p*‐value of <0.05 was considered statistically significant.

## 3 RESULTS

### 3.1 Isolation and identification of AKK bacteria from musk deer

**FIGURE 1:**
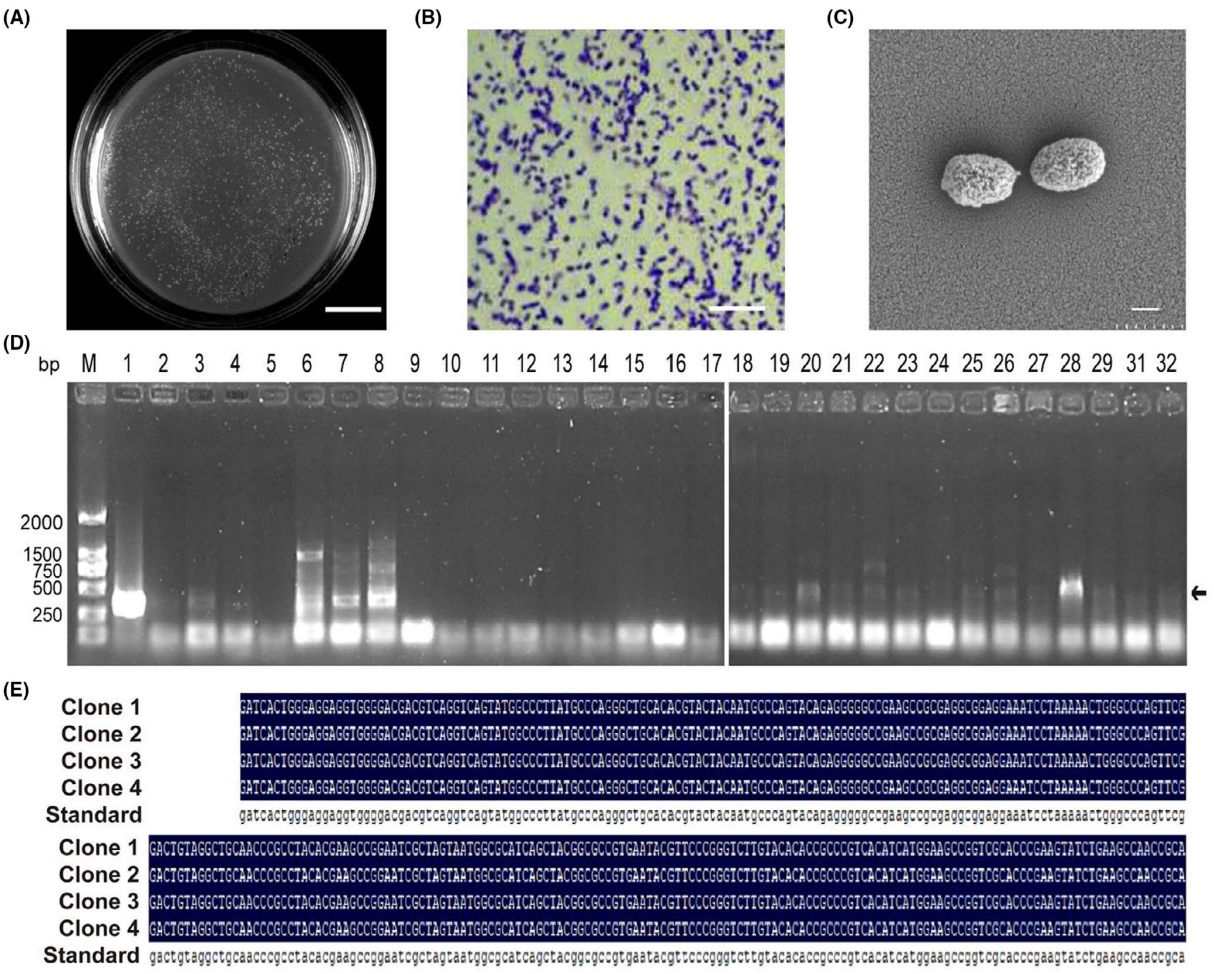
Morphologic observation of AKK isolated from musk deer. (A) colony morphology; (B) gram staining; (C) scanning electron microscopy. Morphology of the AKK bacteria was observed under an optical microscope (×1000) and under a scanning electron microscope (×10 000). (D) Identification of suspected AKK‐D by PCR. The PCR gel electrophoresis of suspected AKK‐D. Lane M: DL2000 DNA Marker. All the other lanes are the PCR products from musk deer fecal samples. (E) Results of DNA sequencing of specific primers for PCR of suspected AKK‐D.

AKK is a specific commensal bacterium in human intestinal mucosa and is used in next‐generation probiotics related to obesity, diabetes and other diseases. However, the low abundance of AKK and the difficulty of its in vitro culture make isolating new strains difficult. The safest strategy for developing probiotics for musk deer protection would be to use autologous intestinal bacteria. This would also allow AKK bacteria from different sources to be compared for their advantages and disadvantages. Therefore, in this study, we used an improved enrichment medium combined with real‐time quantitative PCR (qPCR) to isolate AKK from 20 musk deer fecal samples (Figure 1A). After systematically optimizing the separation effect, we isolated and purified one suspected AKK strain from three positive fecal samples (Figure 1D). After a preliminary identification by gram staining (Figure 1B), and observation of the morphology under a scanning electron microscope (Figure 1C), the candidate strain was further confirmed by 16S rDNA sequencing and AKK specific PCR amplification. One strain was finally identified as musk deer‐originated AKK (AKK‐D; Figure 1E).

### 3.2 Characterization of the probiotic functions of AKK‐D

**FIGURE 2:**
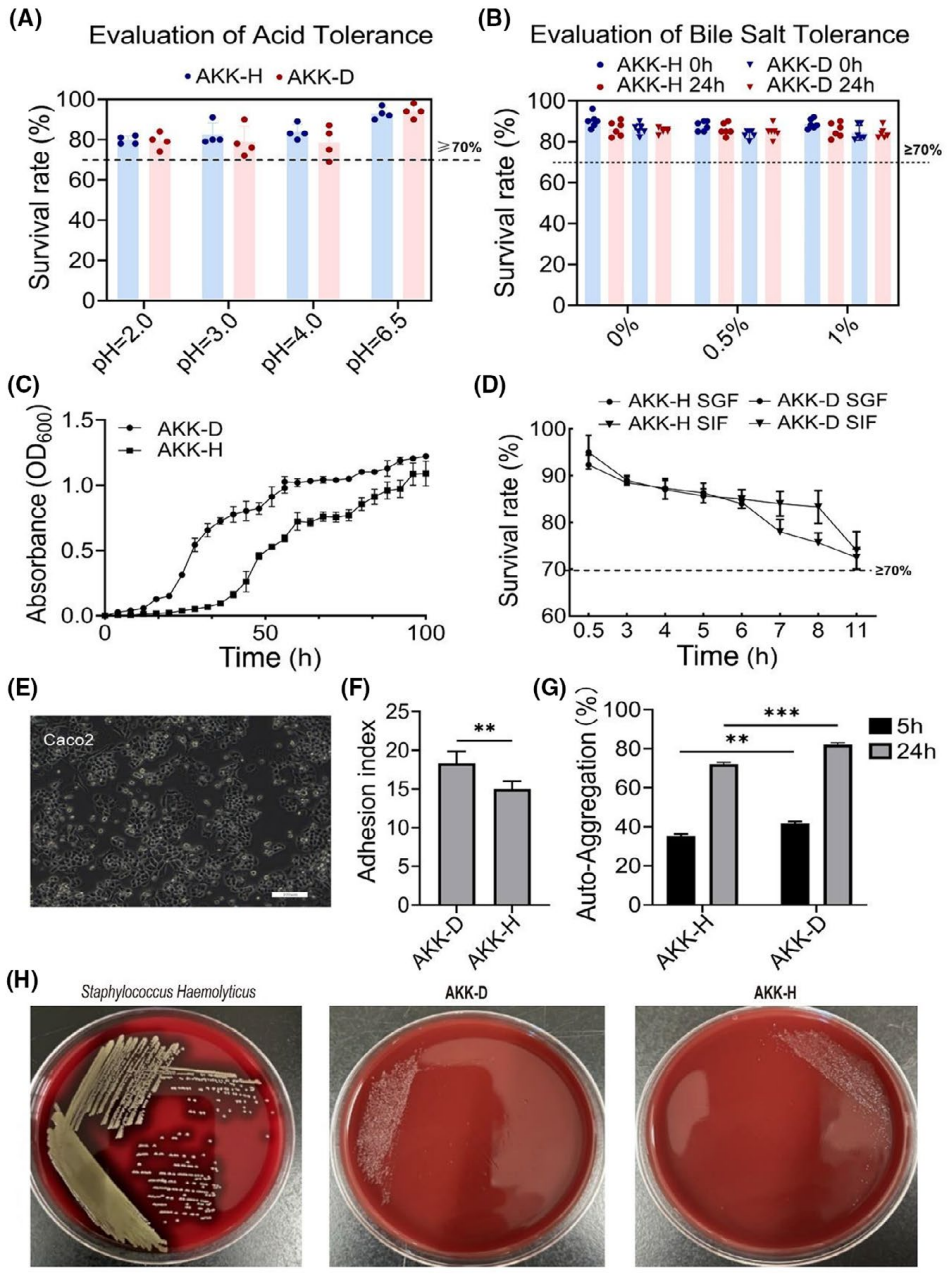
Summary of isolation and characterization of AKK‐D and evaluation of probiotic potential. (A) Survival rate of isolated AKK‐D under acidic conditions for 24 h. (B) Survival rates of isolated AKK‐D in 0.3% and 0.5% bile salt conditions for 4 h. (C) The growth curve of AKK‐D. (D) Tolerance of AKK‐D to simulated gastrointestinal fluids; SGF: Simulated gastric fluid; SIF: simulated intestinal fluid. (E) Microscopic morphology of Caco‐2 in its normal state (×10). (F) Ability of AKK‐D to adhere to Caco2 cells. (G) Auto‐aggregation of AKK‐D. (H) The hemolytic activity of *Staphylococcus Haemolyticus*, AKK‐D, and AKK‐H (from left to right). All data are expressed as mean ± SEM. **p < 0.01 and ***p < 0.001 compared between indicated groups.

A series of in vitro tests were conducted to characterize the probiotic functions of AKK‐D. Results showed that AKK‐H had similar acid and bile salt resistance to AKK‐D, with both strains showeing good acid and bile salt resistance (Figure 2A,B). Regarding reproductive capability, AKK‐D showed a stronger proliferation ability than AKK‐H (Figure 2C). Considering probiotics need to pass through the digestive tract to exert their functions, we measured the resistance of the AKKs to stomach acid and intestinal fluids. As shown in Figure 2D, AKK‐D had a higher tolerance to simulated gastric and intestinal fluids than AKK‐H, but both displayed good tolerance of simulated gastrointestinal fluids. The adhesion ability of the AKK‐D to the IECs was determined by bacterial counting, which revealed an adhesion index of 17.0 ± 3.9, meaning that an average of 17 AKK‐D live bacteria can adhere to each Caco2 cell (Figure 2E,F). The ability of probiotics to adhere to IECs usually determines their ability to colonize the host to reconstitute or maintain the homeostasis of host gut microbiota, and therefore AKK‐D showed stronger probiotic potential than AKK‐H (Figure 2F). AKK‐D also exhibited faster and higher auto‐aggregation and hydrophobicity ability than AKK‐H (Figure 2G, Table 1). Neither of the AKK strains caused hemolysis on the agar blood plates (γ‐hemolytic), indicating that they had no side‐effect on red blood cells (Figure 2H). AKK‐H was classified as weakly adherent in all tested cases, while AKK‐D had a relatively higher aptitude for biofilm formation in a 2.5% glucose solution (Table S2). AKK‐H strain had a high sensitivity to most antibiotics, but was insensitive to penicillin, norfloxacin, and kanamycin, while AKK‐D was highly sensitive to most antibiotics (Table 2). Neither of the strains produced harmful metabolites (Table S3). In summary, our data show that AKK‐D can be used as a potential probiotic strain for further research.

**TABLE 1:** Hydrophobicity assay.

Strains	Contact time (min)	Xylene	Toluene
AKK‐H	15	7.2 ± 0.5	27.9 ± 0.4
	30	39.4 ± 0.3	40.5 ± 0.5
	60	40.3 ± 0.4	45.6 ± 0.4
AKK‐D	15	10.5 ± 0.3	30.9 ± 0.4
	30	45.2 ± 0.3	45.5 ± 0.5
	60	46.3 ± 0.4	48.6 ± 0.3

**TABLE 2:** Results of multidrug resistance of AKK‐D and AKK‐H.

Antibiotics	AKK‐H		AKK‐D	
	Zone of inhibition/mm	Susceptible	Zones of inhibition/mm	Susceptible
Erythromycin	16.5 ± 3.4	H	19.9 ± 3.4	H
Chloramphenicol	20.5 ± 5.6	H	17.5 ± 5.6	H
Amoxicillin	10.4 ± 4.7	I	11.5 ± 4.5	I
Streptomycin	17.5 ± 6.4	H	13.2 ± 6.4	I
Ampicillin	2.5 ± 7.2	L	12.5 ± 7.2	I
Gentamicin	15.6 ± 8.0	H	19.6 ± 8.0	H
Clindamycin	17.3 ± 6.8	H	18.3 ± 6.8	H
Cefotaxime	8.4 ± 7.1	L	12.6 ± 4.4	I
Vancomycin	16.8 ± 3.8	H	20.3 ± 8.0	H
Tetracycline	4.5 ± 8.0	L	12.5 ± 8.0	I
Norfloxacin	1.6 ± 6.8	L	4.7 ± 9.4	L
Kanamycin	0.2 ± 5.3	L	2.5 ± 4.5	L

*Note*: High susceptibility, the zone of inhibition ≥15 mm (H); middle susceptibility, 10–14 mm (I); low susceptibility, ≤10 mm (L).

### 3.3 AKK‐D ameliorates enterotoxigenic *E. coli*‐induced diarrhea in mice

**FIGURE 3:**
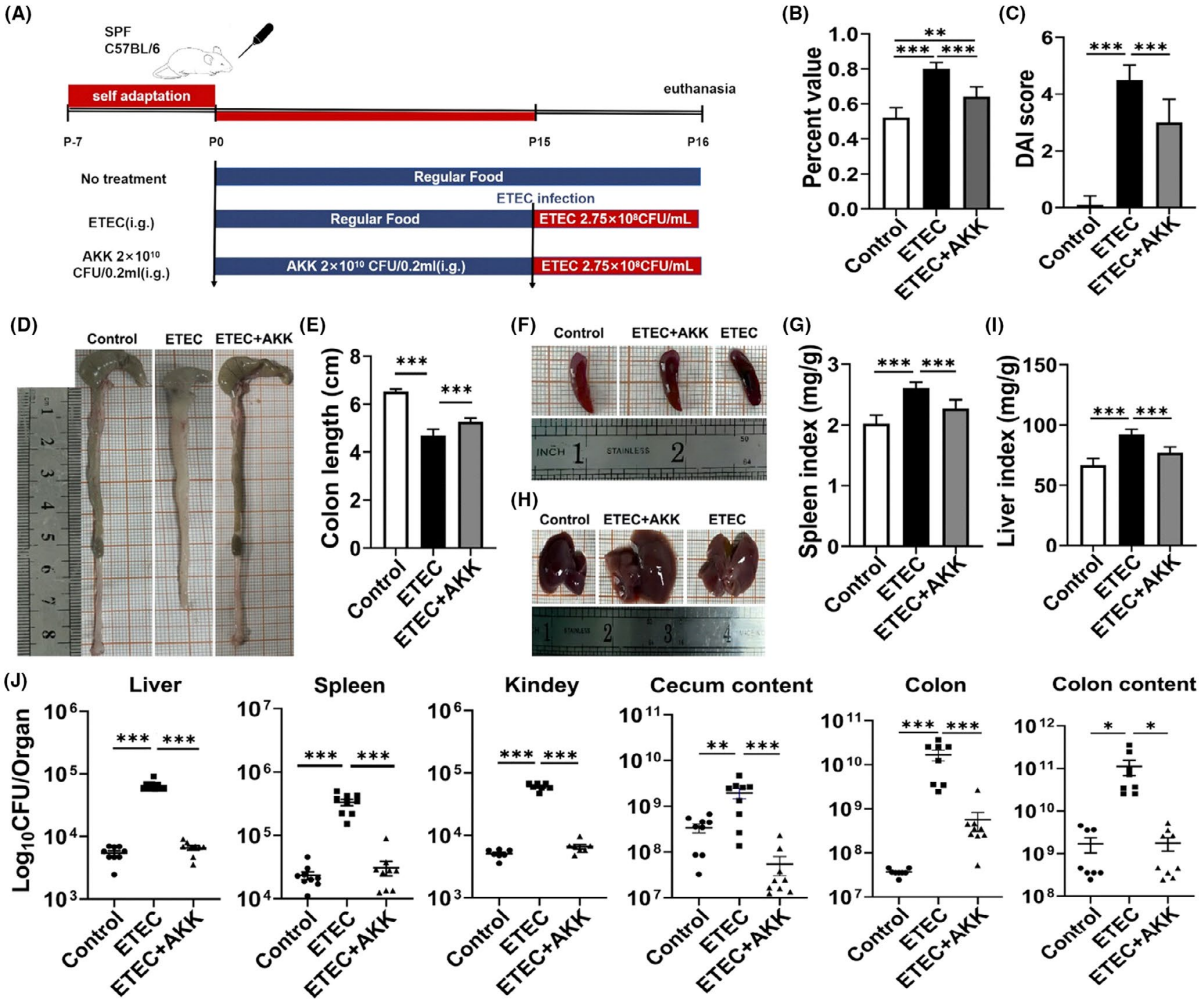
The administration of AKK‐D mitigated ETEC‐induced diarrhea mice. (A) Schematic diagram of experimental process. The mice were euthanized at the end of the experiment to observe the patterns of weight fluctuation and pathogen shedding through feces. (B) The diarrhea indices used to determine the water balance of the gut. (C) DAI score. (D) Macroscopic observation of colon tissues. (E) Colon length. (F) Macroscopic observation of spleen. (G) Spleen index [spleen weight/body weight]. (H) Macroscopic observation of liver. (I) Liver index [liver weight/body weight]. (J) The bacterial loads in various organs and tissues were determined. All data are expressed as mean ± SEM. *p < 0.05, **p < 0.01, and ***p < 0.001 compared between indicated groups.

To evaluate the probiotic effects of AKK‐D *in vivo*, we established a murine diarrhea model and evaluated the protective effects of AKK‐D (Figure 3A). Significantly increased eyelid secretions, unformed feces in the anus (data not shown), fecal water content (Figure 3B), and DAI (Figure 3C) were observed in the model mice, and all these indexes were apparently improved by AKK‐D pre‐administration (*p* < 0.001).

**FIGURE 4:**
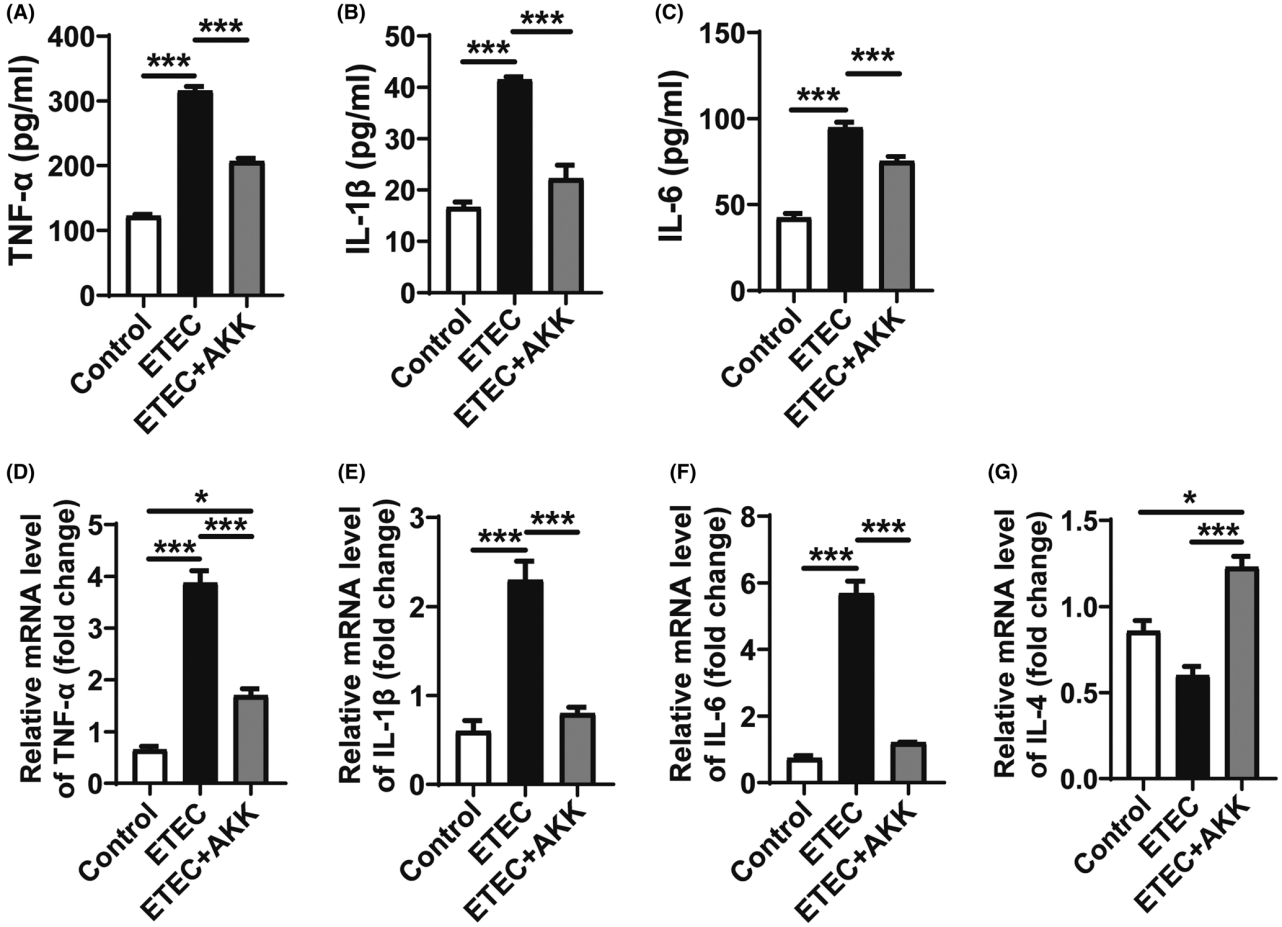
AKK‐D inhibits inflammatory responses in ETEC‐induced diarrhea mice. (A–C) Levels of pro‐inflammatory cytokines TNF‐α (A) IL‐1β (B) and IL‐6 (C) In the sera were determined by qRT‐PCR. (D–G) Levels of pro‐inflammatory cytokines TNF‐α (D), IL‐1β (E) and IL‐6 (F) and anti‐inflammatory cytokine IL‐4 (G) In the colon tissues were determined by qRT‐PCR. Data are presented as mean ± SEM. Statistical analysis was performed by one‐way ANOVA followed by Dunnett's multiple comparison tests. *p < 0.05 and ***p < 0.001 compared between indicated groups.

The diarrhea diseases are predominantly characterized by the phenotype and pathological changes in colon tissue. We found that the colon length of ETEC‐induced diarrhea mice was significantly shortened (Figure 3D,E) and largely restored by AKK‐D. AKK‐D also markedly alleviated spleen swelling and liver enlargement indued by ETEC (Figure 3F–I). A significant increase in ETEC loading in the liver, spleen, kidneys, cecal contents, colon, and colon tissue contents (*p* < 0.01 or *p* < 0.001) was found in diarrhea mice, whereas AKK‐D treatment effectively restored the bacteria loading to normal levels (Figure 3J). Additionally, the inflammatory cytokines IL‐1β, IL‐6, and TNF‐α were upregulated, whereas the anti‐inflammatory cytokine IL‐4 was downregulated at both mRNA and protein levels in the sera and colon tissues of the ETEC mice (Figure 4A–G). In contrast, all these cytokines were reverse regulated by AKK‐D, which indicated that AKK‐D protected the mice from inflammatory responses in diarrhea.

### 3.4 AKK‐D improved disordered intestinal mucosal barrier in diarrhea mice

**FIGURE 5:**
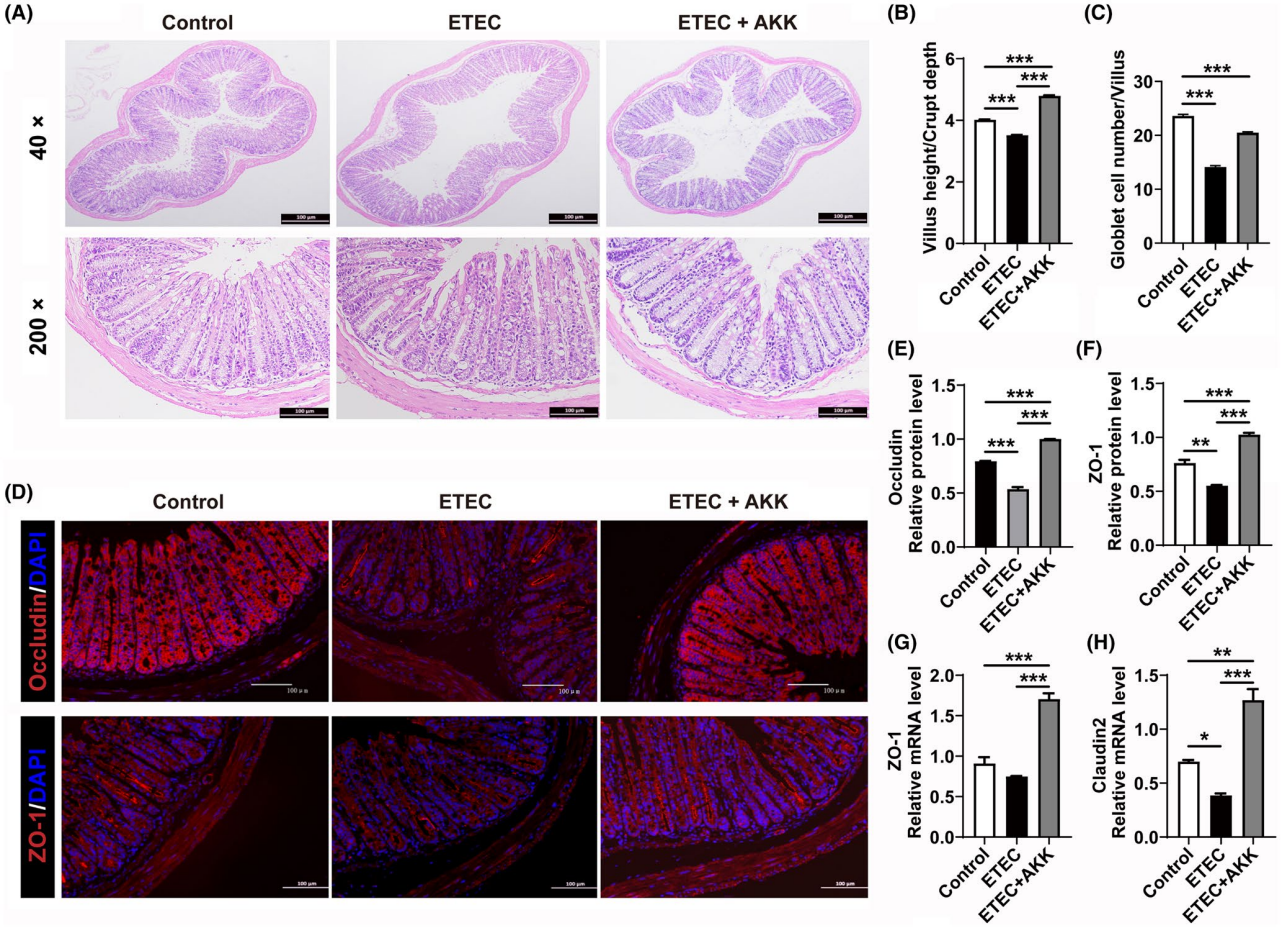
AKK‐D attenuated colon tissue damages and intestinal barrier permeability in ETEC‐induced diarrhea in mice. (A) Representative H&E staining of colon tissues and histological scores. (B) Villus height/Crypt depth ratio. (C) Goblet cell density. (D) Tight junction‐associated proteins were detected by immunofluorescence staining. Bar = 100 μm. (E–F), The relative protein levels of Occludin (E), and ZO‐1 (F). (G), The mRNA level of ZO‐1. (H), The mRNA level of Claudin2. *p < 0.05, **p < 0.01, ***p < 0.001 compared between indicated groups.

The intestinal mucosal barrier is the first line of defense protecting the host from pathogen infections and plays a pivotal role in diarrhea. When H&E staining was performed, decreased goblet cell numbers, atrophy of crypts, and lymphocyte infiltration could be seen in the distal colon of the model mice compared with the normal control (*p* < 0.001), which suggested damage to the intestinal mucus layer and a weakened barrier function. AKK‐D significantly alleviated the pathological damage to colon tissue in the diarrhea mice (Figure 5A–C). Tight junctions (TJs) are one of the most important components of the intestinal barrier, and we therefore measured TJ‐associated protein levels in the colon tissue. As shown in Figure 5D–H, the expression of both Claudin2 and ZO‐1 in the colon of ETEC mice was significantly reduced, while they were significantly upregulated by AKK‐D to an even higher level than in the normal mice. These results collectively indicated that AKK‐D intervention can effectively reduce the severity of ETEC‐induced diarrhea in mice.

### 3.5 AKK‐D maintains intestinal microbiota homeostasis in diarrhea mice

**FIGURE 6:**
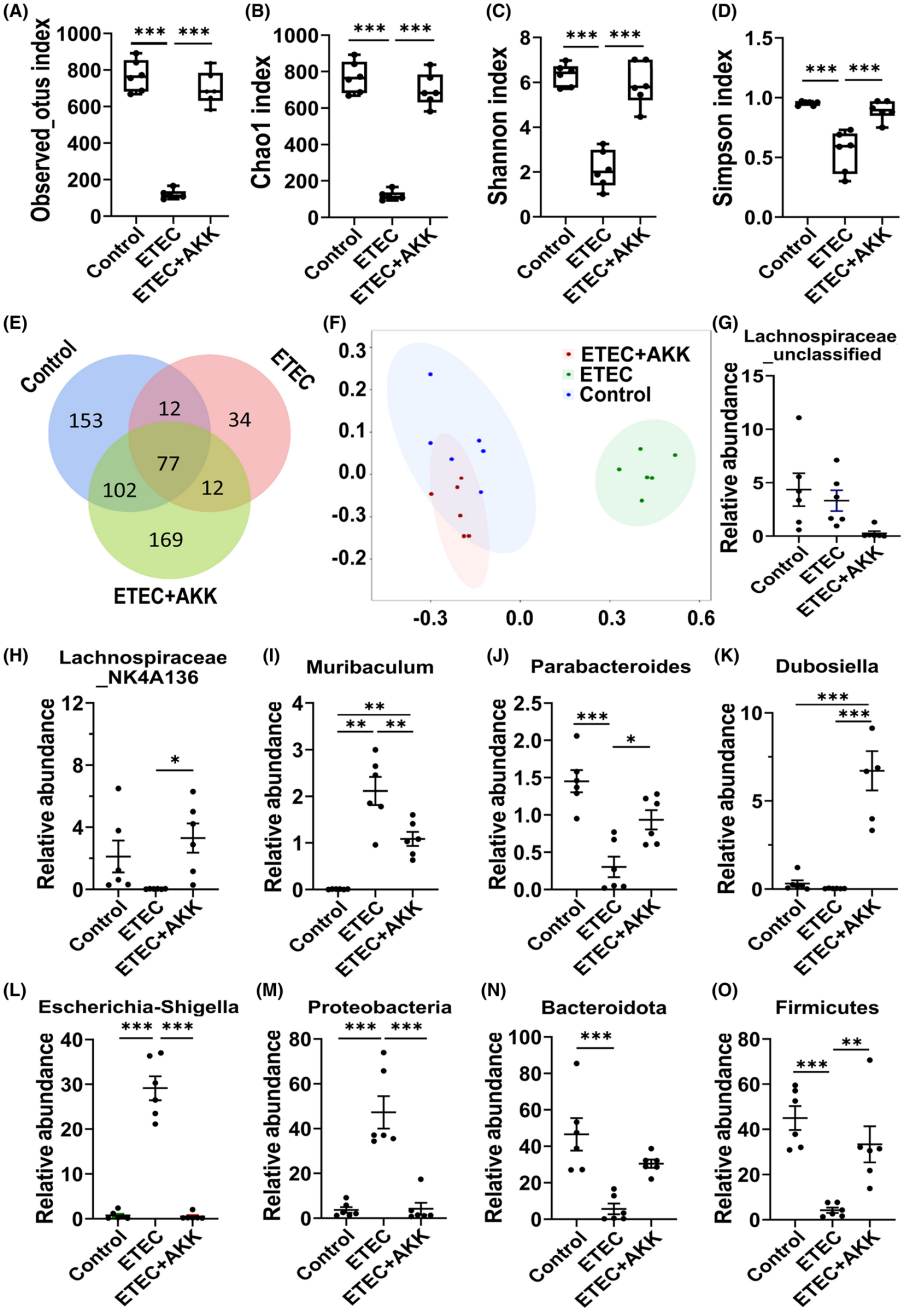
AKK modulated gut microbial community composition in the *Escherichia coli*‐induced diarrhea in mice. (A) Observed_otus index. (B) Chao1 index. (C) Shannon index. (D) Simpson index. (E) Venn diagram. The Venn diagrams show the numbers of OTUs (97% sequence identity) in Control, ETEC, and ETEC + AKK individuals. (F) MDS analysis. (G–L) Difference significance analysis of the genera *Lachnospiraceae_unclassified*, *Lachnospiraceae _NK4A136*, *Muribaculum*, *Parabacteroides*, *Dubosiella*, and *Escherichia‐Shigella* in each group of mice (*n* = 10). (M–O), Relative abundance of the *Proteobacteria* phylum, *Bacteroidetes* phylum, and *Proteobacteria* phylum. *p < 0.05, **p < 0.01, and ***p < 0.001 compared between indicated groups.

Accumulating evidence has suggested that dysregulation of gut microbiota is a key factor in diarrhea. Therefore, we investigated the role of gut microbiota in the alleviation of diarrhea by AKK‐D. We obtained 1 485 145 bacterial 16S rRNA reads from 30 colon content samples, with an average of 82 509 reads per sample. Data analysis uncovered that the α‐diversity significantly decreased in the ETEC group, while AKK‐D intervention significantly increased the α‐diversity of gut microbiota (at least *p* < 0.001, Figure 6A–D). The Venn plot of the ASV distribution represents the number of common and unique ASVs (Figure 6E). In addition, multidimensional scaling (MDS) taxonomic dissimilarity (*β*‐diversity) analysis indicated that the gut microbiota were significantly clustered between the 3 groups at the operational taxonomic units (OUT) level, and the distribution of the AKK‐D‐treated group was closer to and partially overlapped with the control group (Figure 6F), indicating that AKK‐D prevented the gut microbiota imbalance induced by ETEC infection. In addition, there were six significantly different families in these 3 groups: *Muribaculum*, *Paraacteroides*, *Lachnospiraceae_NK4A136*, *Lachnospiraceae_ Unclassified*, *Dubosella*, and *Escherichia Shigella* (Figure 6G–L). Among the top 50 genera, 5 in the ETEC group were significantly upregulated, while 14 were downregulated compared to the control group (*p* < 0.05). However, further analysis at the phylum and genus levels showed that AKK‐D intervention significantly decreased the proportion of *Proteobacteria* (Figure 6M) but increased the proportion of *Bacteroidetes* (Figure 6N) and *Firmicutes* (Figure 6O) compared to the diarrhea model group. The increase in the relative abundance of the probiotics indicated that AKK‐D can increase the abundance of other probiotics, such as *Muribaculaceae*, *Muribaculum*, and *Lachnospiraceae_Unclassified*, thereby increasing its probiotic effects and alleviating the damage to the body's intestinal tissue. These results collectively suggested that reconstitution and maintenance of the homeostasis of gut microbiota may play a critical role in prevention of diarrhea by AKK‐D.

## 4 DISCUSSION

The energy sources and environmental conditions required for the growth and reproduction of AKK are complex, so to achieve efficient isolation of AKK, so in order to clarify the absorption efficiency of various energy sources, to integrate nutrient configurations during the isolation, purification and enrichment processes, and to establish appropriate environmental conditions. Derrien et al. firstly described the isolation and growth medium for the standard strain of AKK (ATCC‐BAA‐835) in 2004,^35^ with bicarbonate buffered medium and mucin basal medium used as base medium and isolation medium, respectively. Plovier and colleagues invented a medium in which mucin was replaced by a combination of glucose, N‐acetylglucosamine, peptone, and threonine,^36^ which can avoid heterologous human compounds while effectively promoting AKK culture. Optimal methods for AKK culture are still being explored. Increases in the scale of production of AKK would not only assist in food, feed or medicine production, but also significantly benefit research on methods of isolating AKK and its relationship with various diseases. At present, isolation of AKK from different animals still presents many problems. While AKK with a high degree of genomic homology with the model strain has been isolated from some animals living in the wild, not nearly enough has been isolated, and the stability and reproducibility of AKK isolates are as yet largely unknown either. More theoretical data are urgently needed to support AKK homology studies.

AKK is now recognized as an important mediator of host metabolism and immunity in several pathogenic environments and has been extensively studied for its health‐promoting benefits, including amelioration of metabolic diseases and promotion of immunotherapy.^37^ However, the mechanism of AKK antagonism in an environment where an ETEC infection is present is currently unknown. In this paper, we investigated the role of musk deer‐derived AKK on the course of ETEC infection. We found that AKK‐D significantly reduced the severity of the diarrhea caused by *E. coli* infection in mice. Consistent with previous studies on AKK‐H, AKK‐D significantly reduced the pro‐inflammatory cytokines like IL⁃1β, IL⁃6, and TNF⁃α, which were elevated in the ETEC‐induced diarrhea model. In addition, AKK‐D promoted intestinal barrier permeability repair via upregulating ETEC‐induced suppression of TJ‐related proteins Occludin, ZO‐1, and Muc2. These results support the notion that AKK‐D prevents infection in part by improving intestinal barrier function.

Intestinal flora can inhibit pathogenic bacteria by competing with them for metabolites and inducing immune responses in the host. It has been reported that diarrhea is closely associated with dysbiosis of the intestinal microflora.^33^ However, the role of gut microbiota in ETEC‐induced diarrhea is still unclear. The present study revealed that the α‐diversity of AKK‐D‐treated and untreated diarrhea mice was significantly different, and AKK‐D improved the reduction in α‐diversity in the intestinal flora caused by ETEC. Further analysis showed a significant increase in the proportions of *Mycobacterium anisopliae* and *Mycobacterium* *thickum* phyla in AKK‐D‐treated mice, indicating that AKK‐D can improve the α‐diversity of the intestinal flora via regulating other probiotic bacteria, thereby increasing their probiotic effects and consequently ameliorating intestinal damage. Undoubtedly, further studies are still needed to investigate the different effects of AKK on the outcome of infection with the same pathogen depending on the environment of the microorganism and the host, and to clarify the quantitative relationship between the roles of AKK in protection of the organism and as a candidate probiotic.

## 5 CONCLUSIONS

This study designed to isolate and screen probiotic strains from forest musk deer and investigate their application is innovative from both theoretical and application perspectives. We showed that AKK of musk deer origin could improve ETEC‐induced diarrhea in mice by regulating the intestinal flora, thus providing a clinical reference for further research on the effects of probiotics on the treatment of intestinal inflammation. The study expands our knowledge of the potential probiotic functions of AKK and lays a solid foundation for future probiotic‐based treatments to improve acute diarrhea.

## AUTHOR CONTRIBUTIONS

Yi Wan and Xiaochang Xue conceived and designed the study; Yan Deng, Yan Wang, Ying Liu, and Xiaoli Yang isolated AKK‐D and validated probiotic effects; Yan Deng and Hai Zhang performed animal experiments; Yan Deng, Yi Wan, and Xiaochang Xue analyzed data, drafted manuscript; all authors revised the final version.

## FUNDING INFORMATION

This research was funded by Shaanxi Administration of Traditional Chinese Medicine Projects (2021‐QYZL‐01, 2021‐QYPT‐001), Key R&D Project in Shaanxi Province (2023‐YBSF‐463), the Foundation of Science and Technology in Shaanxi Province (2020TD‐050), and the Fundamental Research Funds for the Central Universities (GK202205010).

## CONFLICT OF INTEREST STATEMENT

The authors declare no competing financial interest.

## ETHICS STATEMENT

All experiments were approved by the Institute of Animal Use and Care Committee of Shaanxi Normal University (2023–054). The Feng County Huang Forest Musk Deer Breeding Center approved collection of captive musk deer stool samples.

## Supporting information


Tables S1‐S3.
