# Supplementary material for: Akkermansia muciniphila isolated from forest musk deer ameliorates diarrhea in mice via modification of gut microbiota
Source: Animal Model Exp Med. 2024 Jun 3;8(2):295–306. doi: 10.1002/ame2.12441 (PMC11871096; doi:10.1002/ame2.12441)
Supplement: Supplementary file 1 — Tables S1‐S3. [file AME2-8-295-s001.docx]

**Supplementary TABLES**

**TABLE S1** Primer sequences for PCR.

| **Primer** | **Primer sequences** |
| --- | --- |
| IL-6-F | 5’-TGATGCACTTGCAGAAAACA-3’ |
| IL-6-R | 5’-ACCAGAGGAAATTTTCAATAGGC-3’ |
| IL-4-F | 5’-ATCATCGGCATTTTGAACGAGGTC-3’ |
| IL-4-R | 5’-ACCTTGGAAGCCCTACAGACGA-3’ |
| IL-1β-F | 5’-ATGAAAGACGGCACACCCAC-3’ |
| IL-1β-R | 5’-GCTTGTGCTCTGCTTGTGAG-3’ |
| TNF⁃α-F | 5’-ACGTCGTAGCAAACCACCAA-3’ |
| TNF⁃α-R | 5’-TAGCAAATCGGCTGACGGTG-3’ |
| β-actin-F | 5’-GTCGTACCAC AGGCATTGTGATGG-3’ |
| β-actin-R | 5’-GCAATGCCTG GGTAC ATGGTGG-3’ |

**TABLE S2** Biofilm formation ability of AKK-D and AKK-H.

| **Strains** | **TSB 0%** | **TSB 0.25%** | **TSB 1%** | **TSB 2.5%** |
| --- | --- | --- | --- | --- |
| AKK-H | WA | WA | WA | WA |
| AKK-D | WA | WA | WA | MA |

SA, strongly adherent; MA, moderately adherent; WA, weakly adherent.

**TABLE S3** The results of harmful metabolites.

| **Production of biogenic amines** | | **Results** |
| --- | --- | --- |
| Amino acid  decarboxylase test | tyrosine | Medium yellow, negative, no tyramine production |
|  | ornithine | Medium yellow, negative, no putrescine production |
|  | histidine | Medium yellow, negative, no histamine production |
| Nitrate reductase test | | Does not turn blue, negative, does not produce nitrate reductase |
